# Effects of submerged macrophytes (*Elodea nuttallii*) on water quality and microbial communities of largemouth bass (*Micropterus salmoides*) ponds

**DOI:** 10.3389/fmicb.2022.1050699

**Published:** 2023-01-13

**Authors:** Zhijuan Nie, Zhaowei Zheng, Haojun Zhu, Yi Sun, Jun Gao, Jiancao Gao, Pao Xu, Gangchuan Xu

**Affiliations:** ^1^Key Laboratory of Integrated Rice-Fish Farming Ecology, Ministry of Agriculture and Rural Affairs, Freshwater Fisheries Research Center (FFRC), Chinese Academy of Fishery Sciences (CAFS), Wuxi, China; ^2^Wuxi Fisheries College, Nanjing Agricultural University, Wuxi, China

**Keywords:** largemouth bass, ecological aquaculture, Cyanobacterial blooms, bacterial community, submerged macrophytes

## Abstract

Traditional aquaculture ponds are one of the most vulnerable ecosystems; thus, ecological aquaculture is increasingly valued for its beneficial ecological properties and ecosystem services. However, little is known about ecological aquaculture of largemouth bass with submerged vegetation. Here, we designed three ecological ponds of cultured largemouth bass with submerged macrophytes (the EM group) and three ponds with traditional aquaculture (the M group) to reveal the response of water quality, and phytoplankton and bacterial communities, to submerged macrophyte bioremediation during a 90-day culture period. We observed that Cyanobacterial outbreak occurred in the M group ponds from day 7 to the end of the experiment; however, there were no Cyanobacterial blooms in the EM group ponds throughout the culture period. Compared with the M group ponds, the EM group ponds, which had submerged hydrophytes, had significantly decreased concentrations of TP, TN, and COD_Mn_, but significantly increased DO concentrations throughout the experimental period. Moreover, ecological aquaculture with submerged macrophytes showed strong effects on the phytoplankton and bacterial community compositions. In particular, the M group ponds had higher phytoplankton density and mainly included Cyanobacteria, whereas the EM group had lower phytoplankton density and mainly included Chlorophyta. Moreover, higher alpha diversity, as determined by Ace and Simpson index values, was detected for bacterial communities in the EM group ponds. Furthermore, PCoA clearly grouped the bacterial communities according to the two culture modes throughout the culture period. These results indicate that ecological aquaculture with submerged macrophytes can improve water quality, control Cyanobacterial blooms, and affect the diversity and composition of bacterial communities. These valuable effects seem to be beneficial and consistent to maintaining aquaculture ecosystem stability.

## 1. Introduction

Largemouth bass (*Micropterus salmoides*) has been a very economically valuable carnivorous freshwater fish since its introduction from North America to China in the early 1980s (Bai and Li, 2018). Its production reached 620,000 tons in 2020 as a result of intensive pond aquaculture (Fishery and Yearbook, 2021). In the constant pursuit of high yields, the exogenous feed input is continually increasing. Large amounts of unused artificial feed and fish excretion are deposited at the bottom of ponds, and most ponds are small, shallow, and stagnant; this can lead to a series of pollution concerns for aquatic environments, such as eutrophication, water quality deterioration, bloom-forming cyanophyte outbreaks, and diseases of aquaculture products (Munni et al., 2013; Costa et al., 2014; Liu C. et al., 2021). Generally, traditional intensive pond aquaculture easily causes environmental degradation, which can seriously affect the whole pond ecosystem environment and aquaculture output (Tang et al., 2021). Therefore, it is very important to develop a healthy and sustainable aquaculture model for long-term aquaculture development.

Aquatic macrophytes are the main primary producer in shallow, clearwater systems, and their composition and structure can enhance the biodiversity and complexity of aquatic ecosystems (Van Nes et al., 2002; Meerhoff et al., 2003; Choi et al., 2014). Numerous previous studies showed that aquatic macrophytes perform critical ecological functions in aquatic ecosystems, such as providing shelter and food for organisms (Scheffer, 1999; Burks et al., 2001), reducing phytoplankton biomass by allelopathic inhibition (Jeppesen et al., 2000; Amorim and Moura, 2020), removing nutrients from the water, controlling sediment resuspension (Moss et al., 2013; Xiong, 2019), helping improve water quality, and maintaining a clear water state (Wang et al., 2008; Su et al., 2018). Compared with floating plants, submerged macrophytes can directly contact the sediment and absorb the nutrients, which further enhances purification (Strange et al., 2019). *Elodea nuttallii*, as a common submerged macrophyte with strong adaptability (Barrat-Segretain et al., 2002; Megateli et al., 2013; Wegner et al., 2019), is widely used for water restoration and algal growth restrictions in the culture of red swamp crayfish and Chinese mitten crab, can significantly improve the survival rate and growth performance of cultured species (Liu et al., 2014; Vanderstukken et al., 2014; Zeng et al., 2018; Yao et al., 2019). Adsorption and utilization of nitrogen, phosphorus, and other nutrients by aquatic macrophytes has been widely used in lake and river ecosystem restoration and sewage treatment (Megateli et al., 2013; Yu et al., 2019). However, there is still a lack of comprehensive research on *in situ* remediation of pond aquaculture that incorporates submerged plants.

Phytoplankton and microbial communities are essential components of natural ecosystems. As a component of pond habitats, phytoplankton and microbial communities play important roles in the biogeochemical cycling of organic matter and nutrients, and biotransformation of pollutants. Excessive nutrients and an imbalanced microorganism community can result in outbreaks of harmful algal and pathogenic bacteria, which easily accumulate and infect aquatic animals; this results in disease and mass mortalities of aquaculture species. Therefore, a healthy and stable phytoplankton and bacterial ecosystem is an important requirement for producing high yields of healthy aquaculture organisms (Ding et al., 2021). Water with submerged plants will have unavoidable environmental changes, and submerged plants have been demonstrated to be vital to the microorganism community (Van der Gucht et al., 2007). To some degree, plants and microbes have both adapted to using their mutually beneficial close associations (Chao et al., 2021). However, little is known about the interactions between macrophytes and microbes during aquaculture of largemouth bass with submerged vegetation.

The main purpose of this study was to develop a new ecological aquaculture model of largemouth bass to solve the problem of water eutrophication caused by traditional aquaculture. To date, integrated submerged macrophytes aquaculture (ISMA) are proven to be effective in practice (Zeng et al., 2018; Yao et al., 2019), but there has been no comprehensive research. Thus, we applied the ISMA model in largemouth bass aquaculture ponds to reveal the response of water quality, and phytoplankton and bacterial communities to submerged macrophyte bioremediation during a 90-day culture period. We constructed phytoplankton and bacterial molecular ecological networks, and screened biomarkers by differential analysis. Furthermore, we identified the key environmental factors that affected the structure of the microbial community in the water, which can provide theoretical support for new technology based on the ISMA model that leads to healthy aquaculture.

## 2. Materials and methods

### 2.1. Experimental design and management

The experiment was conducted at the Yang Zhong experimental base (Freshwater Fisheries Research Center, CAFS; N32°31′, E119°80′). In this study, the three ponds without submerged macrophytes were considered the control group (M), and the three ponds with submerged macrophytes were considered the experimental group (EM). One month before the start of the experiment *Elodea nuttallii* was collected from Yang Zhong experimental base and planted to three ponds, then, six ponds are filled with the same volume of water (All of which are new ponds with consistent initial conditions). The largemouth bass were obtained from Zhanglin Fishery Co., Ltd. (Anhui, China) and allowed to acclimate to a pond for 2 weeks, and they were fed commercial fish food [≥47% crude protein, ≥5% crude lipid (Xinxin Tianen Aquafeed, Zhejiang, China)]. Subsequently, fish with an initial body weight of 14.50 ± 0.23 g were arbitrarily allocated to the six ponds used in this study (0.17 ha) with the same stocking density of 43.48 g·m^−3^. During the experiment, fish were fed two times daily (08: 00 and 18: 00) to apparent satiation. Three months before the start of the experiment 10 l pond water each pond and stored at 4°C to maintain the bacterioplankton community.

### 2.2. Sample collection and measurement

**Figure 1:**
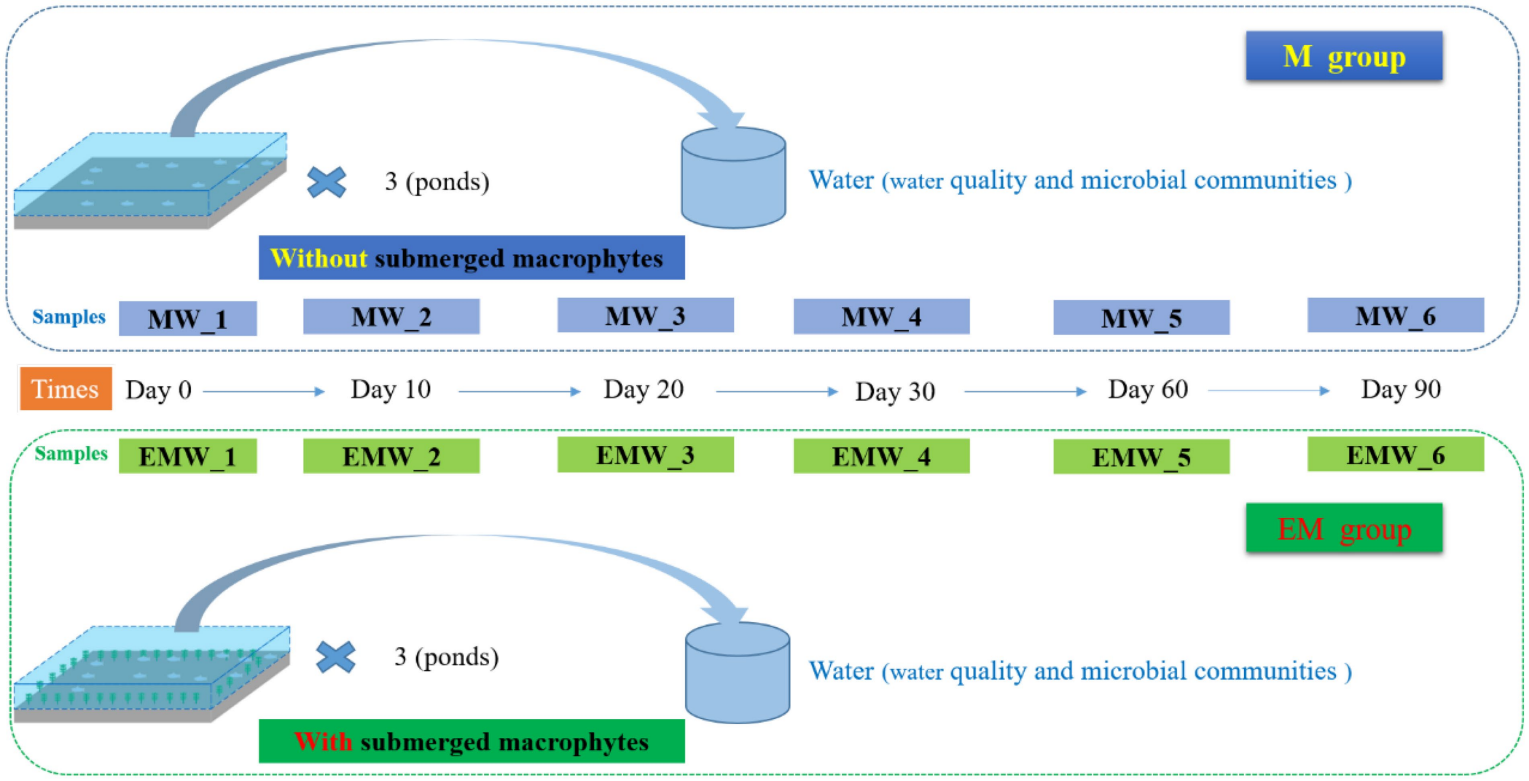
A diagram of the experimental design (timeline).

The experimental period was 90 days (June 28, 2021-September 28, 2021). Thirty fish in each group were collected separately at the beginning and the end of the experiment, and anaesthetized with 100 mg·L^−1^ MS-222, body weight, body length, body thickness and liver and visceral weights of fish were measured. Total of 30 l of water samples were collected by the mixing method of uniform five-point sampling in the same pond on days 0, 10, 20, 30, 60, and 90; and mixed samples of water from the upper, middle, and lower portions of the pond at each point. M group samples were labeled as MW_1, MW_2, MW_3, MW_4, MW_5, and MW_6, respectively, whereas EM group samples were labeled as EMW_1, EMW_2, EMW_3, EMW_4, EMW_5, and EMW_6, respectively (Figure 1). Water dissolved oxygen (DO) concentration, temperature (T), and pH were measured using an HQ30D portable multimeter (HQ30D; HACH, Ames, IA, USA) and YSI Professional Plus system (YSI Inc., YellowSprings, OH, USA) at all sampling points. All water samples were collected and tested from 9: 00 a.m. to 10: 00 a.m. We collected water samples from these points for laboratory analyses. Concentrations of TP, TN, NH_4_^+^-N, NO_2_^−^-N, NO_3_^−^-N, and COD_Mn_ were determined according to standard methods (State EPA of China, 2002). Additionally, the phytoplankton samples were collected by removing 1 L of the mixed water and preserved in Lugol’s iodine solution for further species and quantity determination. Finally, another 500 mL of water from each pond was filtered onto 0.2-μm polycarbonate membranes. Membranes were folded into a 10-mL sample tube with tweezers and then frozen at-80°C until DNA extraction.

In addition, we calculated the weight gain rate (WGR), specific growth rate (SGR), and hepatosomatic somatic index (HSI) as parameters reflecting growth performance, as follows: WGR (%) = (*W*_t_ – *W*_0_) × 100 /*W*_0_, SGR (% day^−1^) = (ln*W*_t_ – ln*W*_0_) × 100/days, HSI (%) = (hepatosomatic weight/body weight) × 100.where *W*_0_ is the initial body weight and *W*_t_ is the final body weight.

### 2.3. Phytoplankton analysis

Phytoplankton counts and species identification were carried out in the laboratory with an optical microscope (Olympus CX31, Japan) and a stereoscope (Olympus SZX10, Japan), respectively. Further calculations were evaluated with the Mcnaughton dominance index (*Y*), *Y* = (n*_i_/N*) *× f_i_* (“*f_i_*”means the frequency of occurrence of species “*i*,” and “*n_i_*” means the number of individuals of species “*i*”; “*N*” means the total number of phytoplankton in the same sample), throughout this study, *Y* > 0.02 was considered a dominant species.

### 2.4. DNA extraction and PCR amplification

DNA was extracted from water membranes using the EZNA™ Water DNA Kit (Omega, USA) following the manufacturer’s protocols. The DNA from all samples was examined for purity by electrophoresis on 1.0% agarose gels, and DNA concentrations were detected by a Nano Drop 2000 spectrophotometer (thermos, USA).

The V3–V4 regions of 16S rRNA of microorganisms were amplified by PCR with primers 338F (5′-ACTCCTACGGGAGGCAGCAG-3′) and 806R (5′-GGACTACHVGGGTWTCTAAT-3′). PCR was conducted in a 20-μL volume, which contained 4 μL 5× FastPfu buffer, 2 μL 2.5 mmol/l dNTPs, 0.8 μL Primer (5 μM), 0.4 μL FastPfu polymerase, and 10 ng DNA template. PCR were performed in triplicate for each sample. The thermocycling conditions were as follows: initial denaturing at 95°C for 3 min; followed by 27 cycles of 30 s at 95°C, 30 s at 55°C and 30 s at 72°C; and final extension at 72°C for 10 min.

### 2.5. MiSeq sequencing and bioinformatics analyses

PCR products were purified with the AxyPrep DNA Gel Extraction Kit (Axygen, USA) following the manufacturer’s instructions. PCR products were then eluted with tris HCl and detected by 2% agarose gel electrophoresis, and detected and quantified using QuantiFluor™-ST (Promega, USA). Subsequently, we purified and constructed the sequencing library. The purified amplified fragments were constructed into PE2*300 library according to the Illumina MiSeq platform (Illumina, USA), and sequenced by the MiSeq PE300 platform (Shanghai Meiji Biomedical Technology Co., Ltd., China).

Bioinformatic analysis of the water microbiota was carried out using the Majorbio Cloud platform^1^. Raw sequences were initially screened and submitted to quality control by Trimmomatic v0.33 over a 50-bp sliding window with the cutoff threshold for average base quality score set to 20. Then, according to the overlap, the sequences at both ends were spliced with a 20% maximum allowed error rate and 80% minimum coverage. Subsequently, based on sequences that overlapped by more than 10 bp and the default maximum allowable error rate, the PE reading was assembled with FLASH v1.2.11. Finally, after using UCHIME v8.1 to identify and remove chimeric sequences, quality-controlled reads were obtained. Effective reads were analyzed with Uparse v7.0.1001, following which all sequences with 97% similarity were clustered to obtain operational taxonomic units (OTUs). The subsequent analyses were based on OTUs. Using Silva as the reference database, the Naive Bayesian classifier with 70% confidence was used for classification. ACE and Simpson indices were used to estimate alpha diversity and evaluate bacterial richness and diversity, and The similarity among the microbial communities in different samples was determined by principal coordinate analysis (PCoA) based on Bray–curtis dissimilarity using Vegan v2.5–3 package. The linear discriminant analysis (LDA) effect size (LEfSe)^2^ was performed to identify the significantly abundant taxa (phylum to genera) of bacteria among the different groups (LDA ≥ 3.5, *p* < 0.05).

### 2.6. Statistical methods and data analysis

Data were collated using Microsoft Excel and all results are presented as the means ± SD values. Student’s t-test was used to analyze differences in water quality, phytoplankton composition, and microbial communities between the two treatments. Differences with *p* < 0.05 between groups were identified as significant, in this experiment, *p*-value generally refers to the difference of two treatments at the same time point. All data were analyzed using IBM SPSS Statistics v26.

## 3. Results

### 3.1. Growth performance of Micropterus salmoides and water quality

The growth performance was significantly divergent between EM and M groups (Table 1). Specifically, the *Micropterus salmoides* in EM group than M group had significantly higher body length, body thickness, body weight, weight gain rate (WGR), and specific growth rate (SGR; *p* < 0.05). Whereas fish in the EM group showed a significantly lower HSI than those in the M group (*p* < 0.05).

**Table 1:** Growth performance indices of largemouth bass.

Growth performance indices	From EM group	From M group
BL/mm	221.15 ± 7.67*	207.13 ± 8.06
BT/mm	36.26 ± 2.00*	34.51 ± 2.89
BW/g	252.86 ± 22.21*	214.99 ± 25.37
WGR/%	1668.26 ± 155.34*	1403.41 ± 177.40
SGR/(%/d)	3.19 ± 0.10*	3.00 ± 0.13
HSI/%	1.59 ± 0.15	2.09 ± 0.20*

*p* < 0.05 indicated significant (*). BL, Body length; BT, Body thickness; BW, Body weight; WGR, Weight gain rate; SGR, Specific growth rate; HSI, Hepatosomatic somatic index.

The physicochemical properties of water samples are shown in Table 2. During the 3-month trial period, there were not significant differences in water temperature between EM and M groups, which ranged from 26.8°C to 32.3°C, whereas the other parameters showed significant dissimilarities. Most obviously, compared with the M group ponds, the EM group ponds with submerged hydrophytes had significantly decreased concentrations of TP, TN, and COD_Mn_ (*p* < 0.05), but significantly increased DO concentrations on day 0, 10, 20, 60 and 90 (*p* < 0.05). NH_4_^+^-N in the EM group ponds was significantly lower than that in the M group ponds on day 10 (*p* < 0.05); however, in other periods, NH_4_^+^-N was more similar and the concentrations were all less than 0.2 mg/l. NO_2_^−^-N and NO_3_^−^-N in the EM group ponds were significantly higher than those in the M group ponds on days 20, and 30 (*p* < 0.05), but there were no significant differences on days 60 and 90 (*p* > 0.05).

**Table 2:** Physicochemical parameters of water in M and EM groups.

Time (d)	Group	Temperature (°C)	pH	TP (mg/L)	TN (mg/L)	NH_4_^+^-N (mg/L)	NO_2_^—^N (mg/L)	NO_3_^—^N (mg/L)	DO (mg/L)	COD_Mn_ (mg/L)
0	M	27.07 ± 0.31	7.57 ± 0.21	0.412 ± 0.026	2.262 ± 0.109	0.241 ± 0.038	0.024 ± 0.001	0.412 ± 0.054	5.43 ± 0.25	14.419 ± 0.712
	EM	27.10 ± 1.65	9.23 ± 0.21	0.409 ± 0.027	2.247 ± 0.108	0.187 ± 0.022	0.007 ± 0.002	0.500 ± 0.043	10.84 ± 0.41	11.115 ± 1.400
	*p*-value	0.976	**0.001**	0.904	0.881	0.099	**<0.001**	0.093	**<0.001**	**0.022**
10	M	28.47 ± 0.31	8.43 ± 0.06	0.919 ± 0.012	2.562 ± 0.117	0.434 ± 0.067	0.016 ± 0.001	0.006 ± 0.005	6.11 ± 0.26	12.204 ± 0.351
	EM	28.47 ± 0.15	9.03 ± 0.15	0.518 ± 0.013	1.737 ± 0.026	0.198 ± 0.014	0.028 ± 0.008	0.312 ± 0.082	8.64 ± 0.65	11.153 ± 0.117
	*p*-value	1.000	**0.009**	**<0.001**	**<0.001**	**0.004**	0.058	**0.003**	**0.003**	**0.008**
20	M	31.87 ± 0.45	8.60 ± 0.20	1.034 ± 0.021	1.791 ± 0.232	0.071 ± 0.027	0.003 ± 0.001	0.650 ± 0.008	7.15 ± 0.97	11.449 ± 0.237
	EM	31.67 ± 0.21	9.07 ± 0.25	0.482 ± 0.039	1.256 ± 0.051	0.124 ± 0.023	0.010 ± 0.001	0.715 ± 0.030	9.85 ± 0.31	9.588 ± 0.237
	*p*-value	0.524	0.066	**<0.001**	**0.018**	0.056	**0.001**	**0.023**	**0.010**	**0.001**
30	M	28.57 ± 0.06	8.40 ± 0.10	0.516 ± 0.026	2.128 ± 0.176	0.117 ± 0.017	0.013 ± 0.001	0.121 ± 0.063	8.15 ± 0.42	11.025 ± 0.034
	EM	28.60 ± 0.10	9.40 ± 0.53	0.329 ± 0.013	1.687 ± 0.089	0.123 ± 0.019	0.017 ± 0.001	0.268 ± 0.061	8.84 ± 0.46	9.814 ± 0.002
	*p*-value	0.815	**0.032**	**<0.001**	**0.018**	0.731	**0.001**	**0.043**	0.128	**<0.001**
60	M	29.77 ± 0.15	8.53 ± 0.06	0.567 ± 0.009	3.665 ± 0.118	0.039 ± 0.023	0.005 ± 0.002	0.244 ± 0.064	8.63 ± 0.30	10.601 ± 0.172
	EM	29.97 ± 0.21	8.63 ± 0.15	0.320 ± 0.029	2.578 ± 0.569	0.083 ± 0.057	0.009 ± 0.002	0.315 ± 0.026	10.02 ± 0.36	10.040 ± 0.234
	*p*-value	0.251	0.468	**<0.001**	**0.032**	0.289	0.055	0.151	**0.006**	**0.028**
90	M	30.73 ± 0.06	8.63 ± 0.12	0.533 ± 0.005	8.127 ± 0.392	0.166 ± 0.009	0.054 ± 0.011	0.938 ± 0.029	8.12 ± 0.30	11.954 ± 0.287
	EM	30.87 ± 0.31	8.73 ± 0.15	0.132 ± 0.017	5.415 ± 0.165	0.368 ± 0.030	0.051 ± 0.003	1.083 ± 0.098	9.42 ± 0.43	10.528 ± 0.459
	*p*-value	0.566	0.468	**<0.001**	**<0.001**	**<0.001**	0.734	0.07	**0.013**	**0.010**

Values are presented as the means ± SD (*n* = 3). TP, Total phosphorus; TN, Total nitrogen; DO, Dissolved oxygen; COD_Mn_, Chemical oxygen demand.

### 3.2. Water phytoplankton analysis

#### 3.2.1. Cyanobacterial blooms

**Figure 2:**
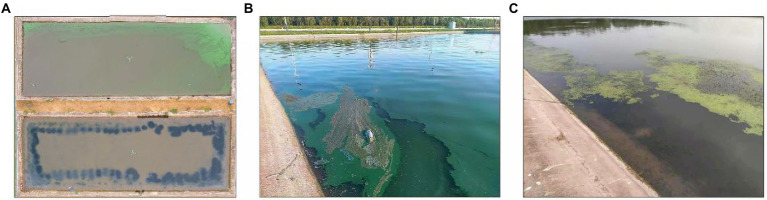
Examples of ponds during aquaculture. **(A)** Top views of an M group pond (at the upper part of the picture) and an EM group pond (lower part of the picture). **(B)** Close-up view of an M group pond. **(C)** Close-up view of an EM group pond.

During the culture experiment, a Cyanobacterial outbreak occurred in the M group ponds from day 7 to the end of the experiment (Figures 2A,B). However, no Cyanobacterial bloom was found in the EM group ponds throughout the culture period (Figures 2A,C).

#### 3.2.2. Community composition

**Figure 3:**
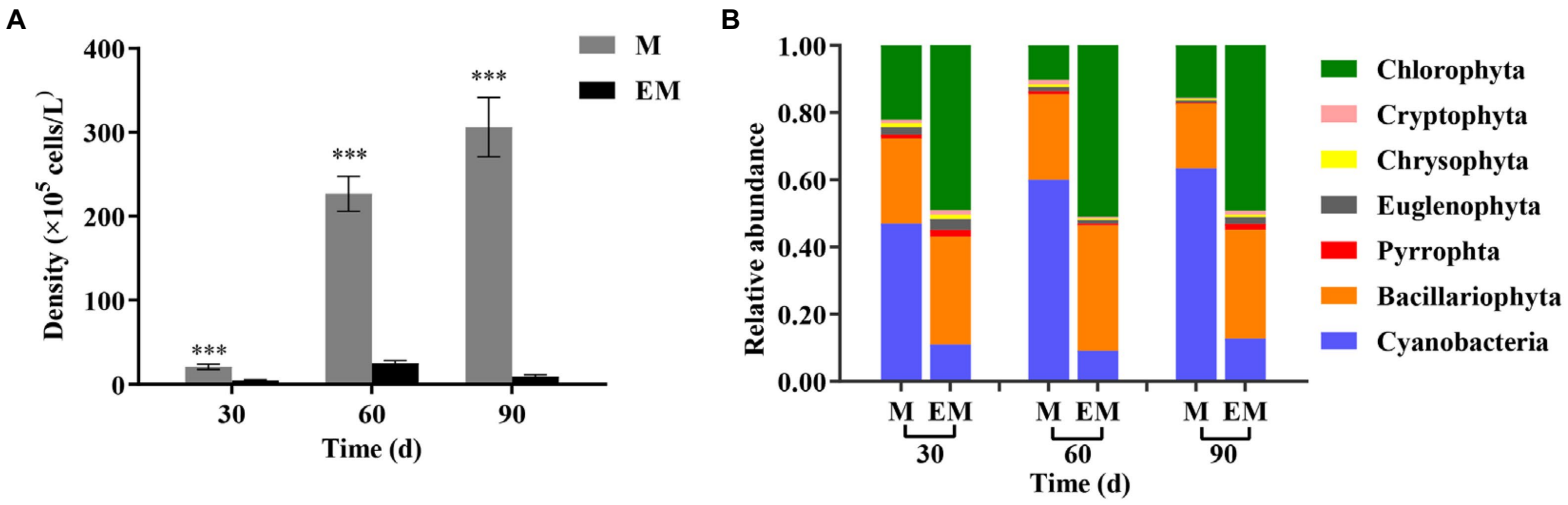
Monthly variations in phytoplankton in M and EM groups. **(A)** Density and **(B)** relative abundance (phylum level). Error bars indicate the standard deviation of the average value. *p < 0.05; **p < 0.01; ***p < 0.001.

The phytoplankton density of the M group increased from 2.07 × 10^6^ to 3.06 × 10^7^ cells/L, which was extremely significantly higher than that of the EM group for every month in the experimental period (Figure 3A). The phytoplankton density of the EM group was only 9.21 × 10^5^ cells/L on day 90. Chlorophyta, Bacillariophyta and Cyanobacteria were the dominant phyla in both groups (Figure 3B). Overall, the M group had higher phytoplankton density and mainly included Cyanobacteria, whereas the EM group had lower phytoplankton density and mainly included Chlorophyta. Further analysis of the dominance index (*Y*) showed that genera *Chroococcus* and *Microcystis*, which are cyanobateria, had total values over 50% in the M group, whereas genera *Chlorella* and *Scenedesmus*, which belong to Chlorophyta, had values close to 50% in the EM group (Table 3).

**Table 3:** Dominant genera and their dominance values of phytoplankton in M and EM groups.

Genus	M			EM		
	Day 30	Day 60	Day 90	Day 30	Day 60	Day 90
*Chroococcus*	0.33	0.37	0.42			
*Microcystis*	0.18	0.12	0.16			
*Merismopedia*			0.02			
*Aphanizomenon*			0.02			
*Melosira*	0.1	0.15	0.04	0.16	0.16	0.17
*Synedra*		0.05	0.03	0.02	0.02	0.04
*Cymbella*	0.04			0.02	0.03	
*Navicula*					0.04	0.02
*Rhizosolenia*			0.02			0.03
*Cocconeis*			0.02			
*Cyclotella*						0.02
*Euglena*				0.02		
*Scenedesmus*	0.04	0.02		0.22	0.26	0.29
*Chlamydomonas*			0.03		0.03	0.09
*Cosmarium*	0.03	0.03		0.04	0.02	
*Ankistrodesmus*			0.02		0.03	
*Chlorella*	0.04		0.03	0.19	0.17	0.18
*Tetraedron*					0.02	
*Chodatella*						0.02

### 3.3. Water microbiota analysis

#### 3.3.1. Sequences profile and alpha diversity

**Figure 4:**
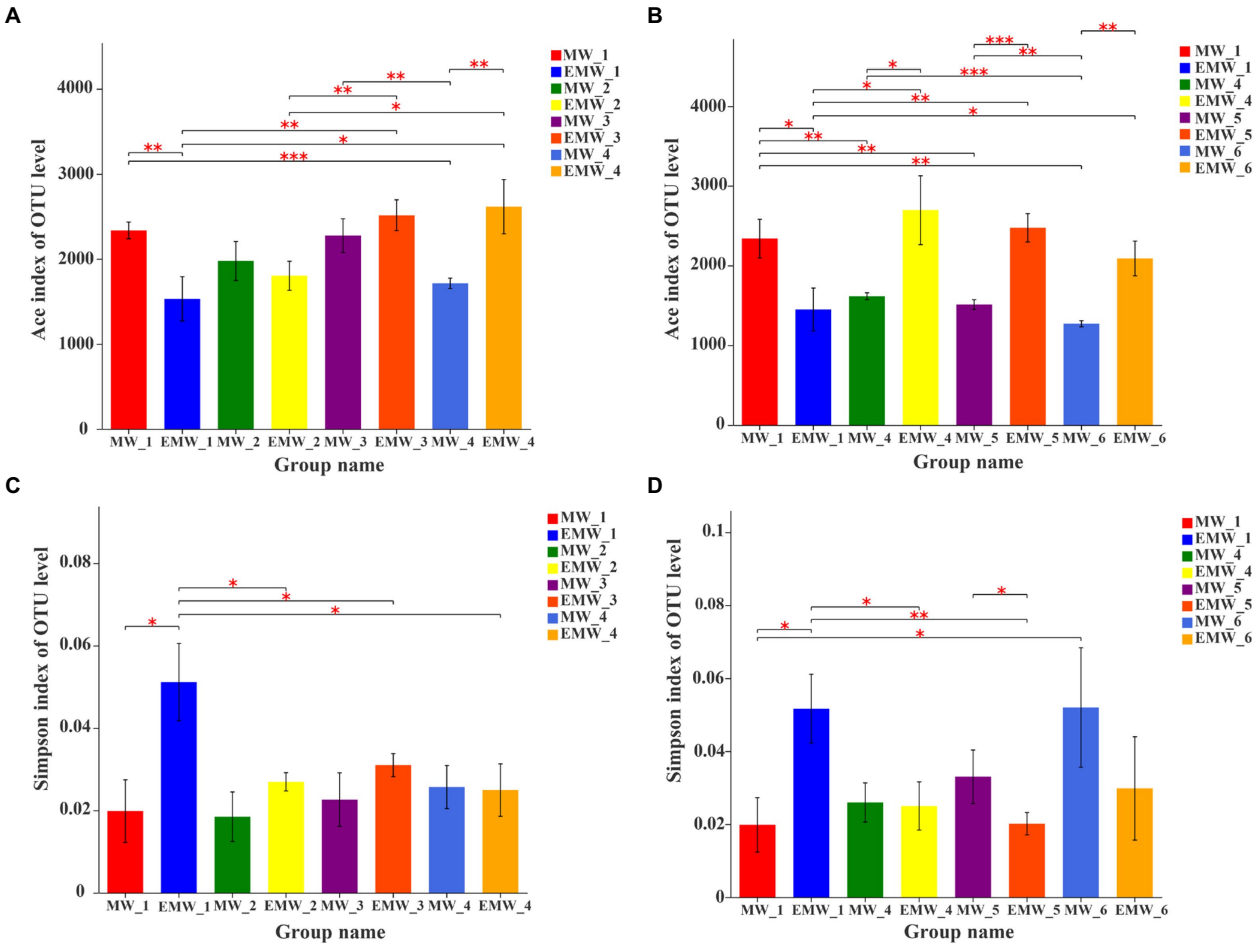
Alpha diversity estimates of the microbial communities at the OTU level in M and EM groups. **(A)** ACE index (the first month), **(B)** ACE index (3 months), **(C)** Simpson index (the first month), and **(D)** Simpson index (3 months). *p < 0.05; **p < 0.01; ***p < 0.001.

In total, 5,579 OTUs with 1,239,660 high-quality 16S rRNA gene sequences were obtained in this study, which were affiliated with 54 phyla, 148 classes, 370 orders, 614 families, 1,223 genera, and 2,261 species. At the beginning of the experiment, the Ace index of the EM group was significantly lower than that of the M group (*p* < 0.05; Figure 4A), but it continued to increase after 1 month, and was significantly higher than that of M group until day 30 (*p* < 0.05). Subsequently, the Ace index of the EM group was similar on days 60 and 90, but it was also significantly higher than that of the M group in the same sampling period (*p* < 0.05; Figure 4B). The Simpson index showed that the diversity of the EM group was significantly lower than that of the M group at the beginning of the experiment (*p* < 0.05), significantly increased from day 10 (Figure 4C), and was finally significantly higher than that of the M group on day 60 (*p* < 0.05). However, there were no dissimilarities between the two groups on day 90 (Figure 4D).

#### 3.3.2. Microbial community composition

**Figure 5:**
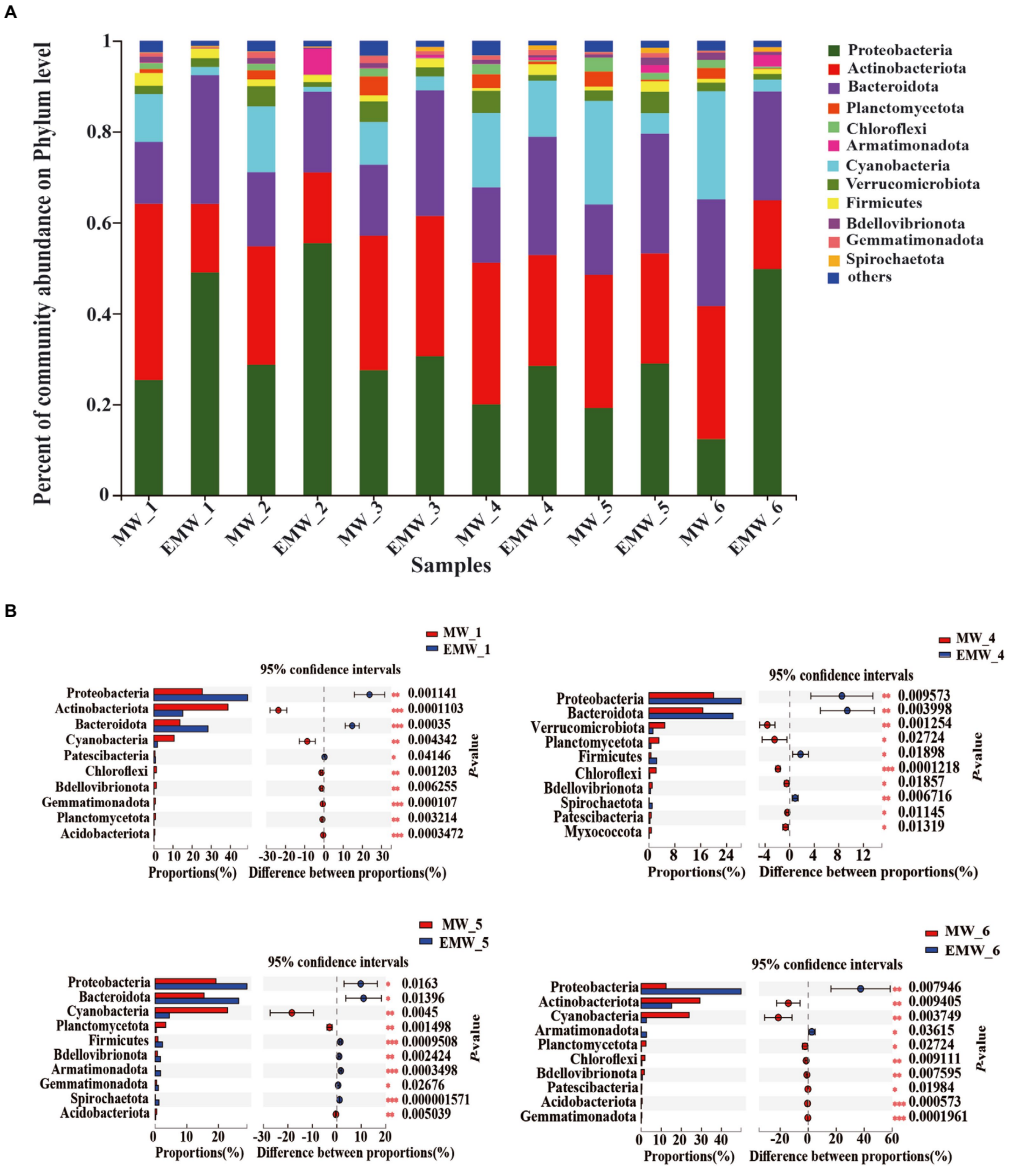
Phylum-level microbial community analysis. **(A)** Percent of community abundance. **(B)** Wilcoxon tests followed by Bonferroni corrections were performed between the two groups on days 0, 30, 60, and 90. Only the 10 most abundant phyla with significant differences are shown; *p < 0.05; **p < 0.01; ***p < 0.001.

The phylum-level classification of these correlated microbial communities were shown in Figure 5A. Proteobacteria, Actinobacteria, Bacteroidetes, Cyanobacteria, and Verrucomicrobia were the dominant phyla in the two groups, and accounted for more than 85% of the total sequences (Figure 5A). Nonetheless, there were also significant differences phyla between the two groups on days 0, 30, 60, and 90 (Figure 5B). On days 0, the dominant phyla in the M group were Actinobacteria (38.82%), Proteobacteria (25.34%), Bacteroidetes (13.61%), and Cyanobacteria (10.56%), whereas the dominant phyla the EM group were Proteobacteria (49.05%), Bacteroidetes (28.31%), Actinobacteria (15.08%), and Firmicutes (2.12%). In particular, Proteobacteria always maintained the highest abundance in the EM group, which was significantly higher than that in the M group. However, the content of Cyanobacteria in the M group was always high and significantly increased, approaching 30% on days 60 and 90, which was significantly higher than that in the EM group. In addition, Bacteroidetes in the EM group were significantly higher than those in the M group on days 30 and 60.

#### 3.3.3. Variations in bacterial community

**Figure 6:**
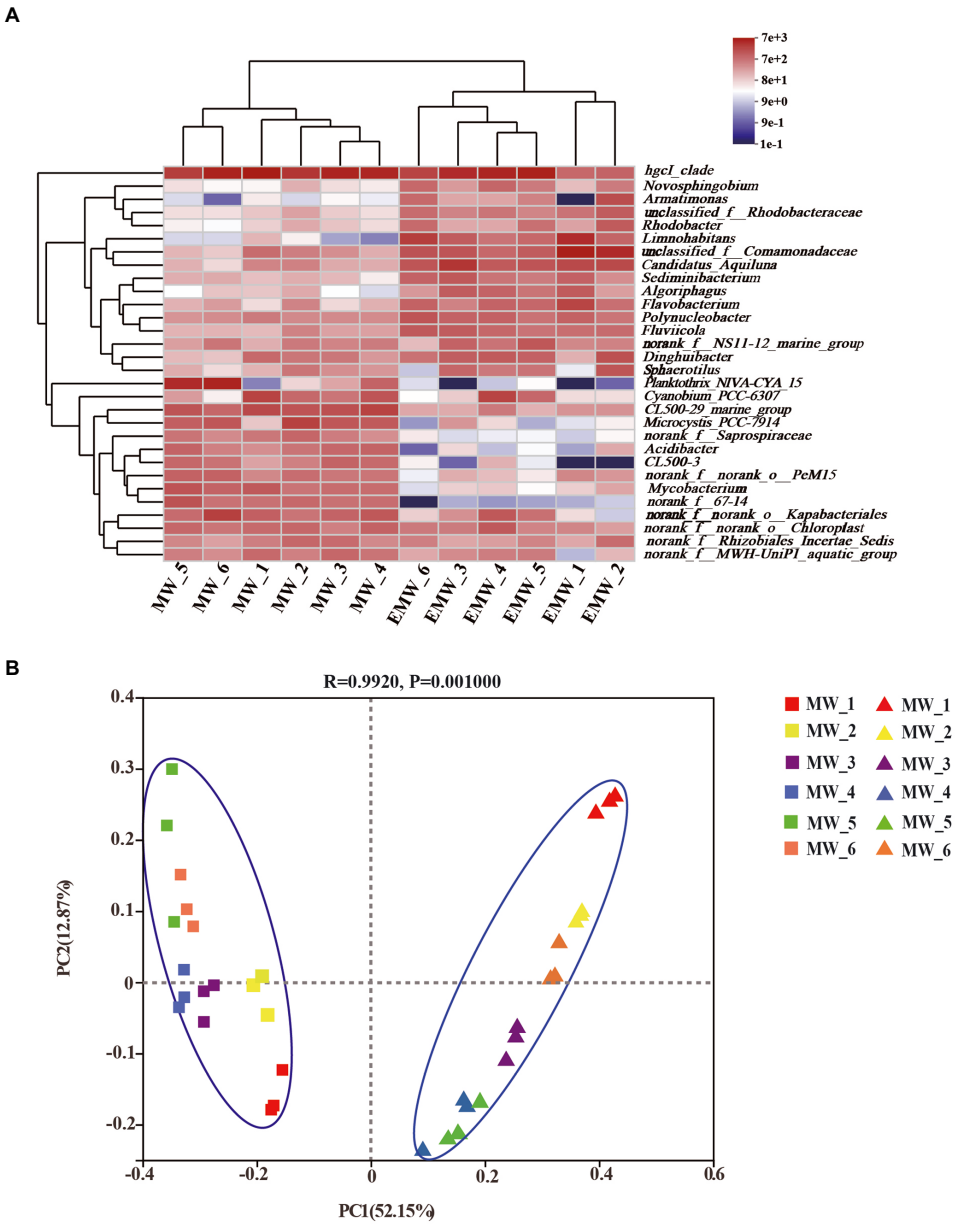
Microbial community analysis at the genus level. **(A)** Heatmap of the relative abundance. **(B)** PCoA results.

Variations in bacterial abundance at the genus level are shown as a heatmap in Figure 6A. The result showed that samples from different periods within each group clustered together; this demonstrated higher similarity of the samples within each group than among groups, and revealed the bacterial genus-level compositions in the water in which largemouth bass were cultured. The PCoA results showed that the first two axes (PC1 and PC2) explained 52.15% and 12.87% of the variance (Figure 6B), respectively, in all samples, and clearly grouped the bacterial communities according to the two culture modes of largemouth bass throughout the culture period. Importantly, the effects of submerged macrophytes on the bacterial community were substantial and stable, and exceeded the differences that resulted from culture time.

**Figure 7:**
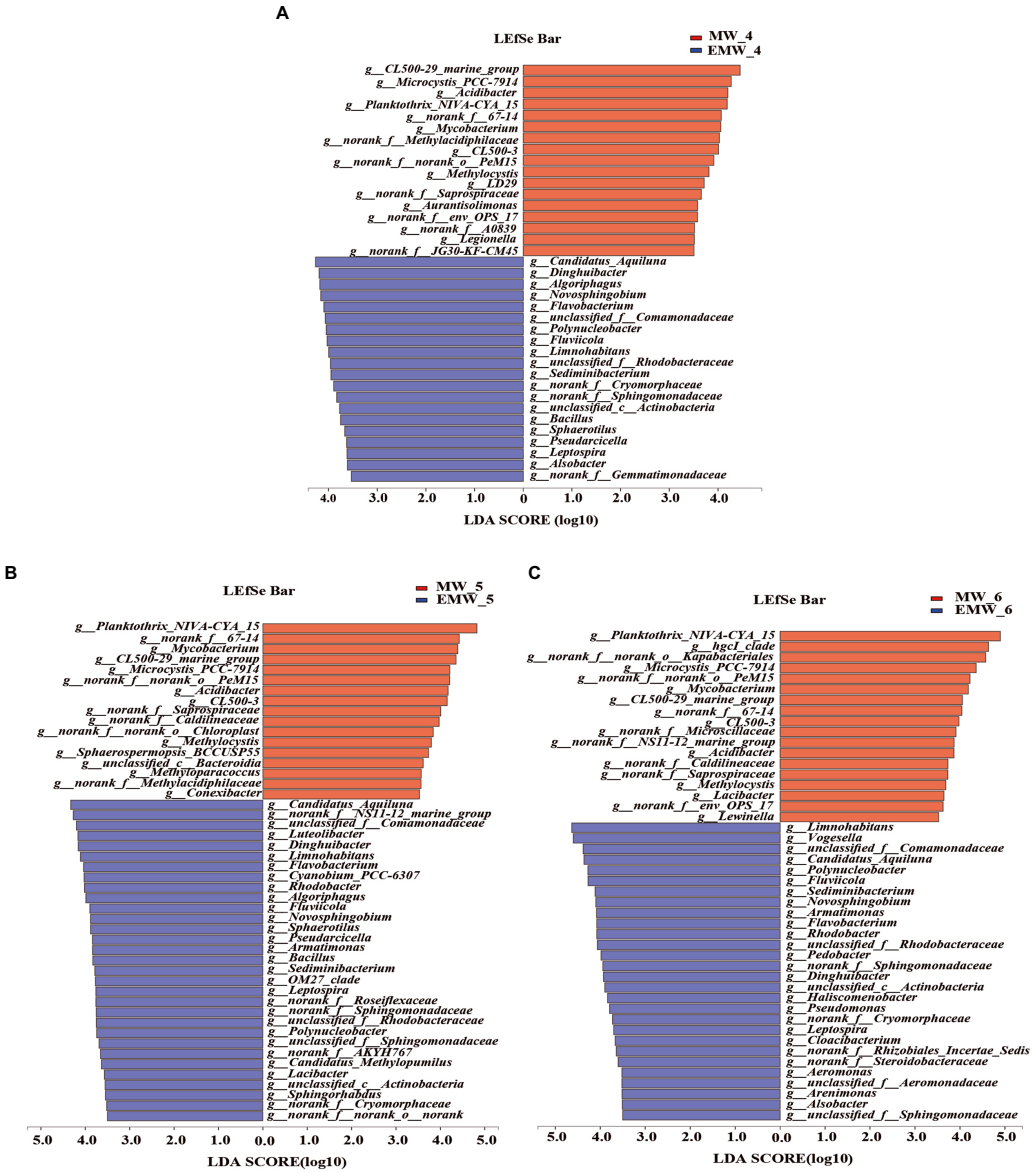
Microbial community analysis at the genus level. Effect size (LEfSe) analyses on days 30(7A), 60(7B), and 90(7C). For taxa, which were defined as unclassified, norank, the name of a higher taxon level was added before its taxon abbreviation (c, class; o, order; f, family).

**Figure 8:**
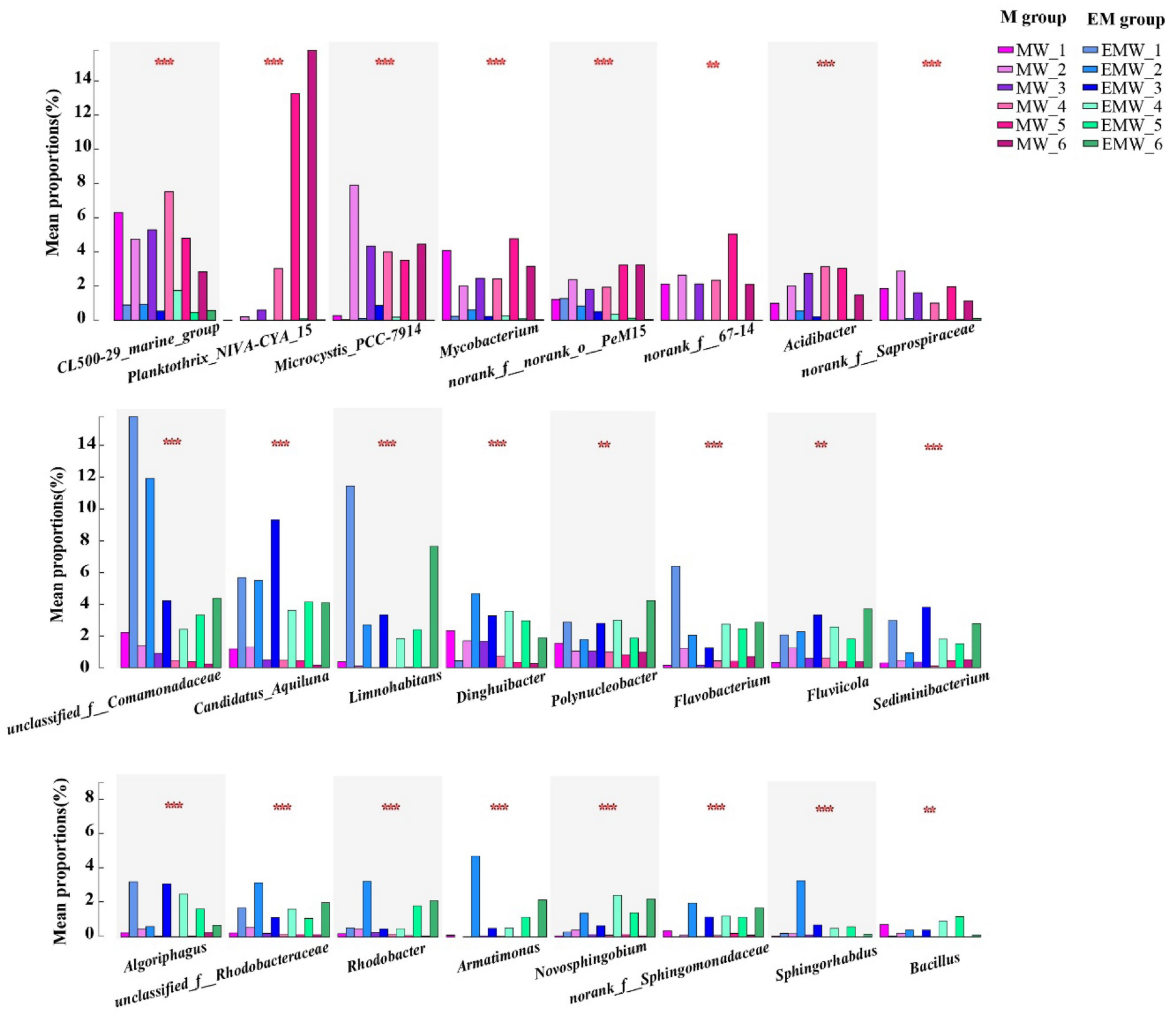
Statistical comparative analysis of relative abundance. *p < 0.05; **p < 0.01; ***p < 0.001.

To further analyze the differential bacterial genera, LEfSe analysis showed over 40 bacterial biomarkers in the two groups; moreover, the number of biomarkers in the EM group was higher than that in the M group (day 30: EM/M = 20/16; day 60: EM/M = 31/17; day 90: EM/M = 28/18; Figure 7). *CL500-29_marine_group*, *Planktothrix_NIVA-CYA_15*, *Microcystis_PCC-7,914*, *Mycobacterium*, *norank_f__norank_o__PeM15*, *norank_f__67–14*, *Acidibacter*, and *norank_f__Saprospiraceae* were clearly biomarkers in the M group, whereas *unclassified_f__Comamonadaceae, Candidatus_Aquiluna, Limnohabitans, Dinghuibacter, Polynucleobacter, Flavobacterium, Fluviicola, Sediminibacterium, Algoriphagus, unclassified_f__Rhodobacteraceae, Rhodobacter, Armatimonas, Novosphingobium*, *norank_f__Sphingomonadaceae, Sphingorhabdus,* and *Bacillus* were biomarkers in the EM group. Interestingly, this result was further supported by statistical comparative analysis of relative abundance (Figure 8). Specifically, eight bacterial biomarkers in the M group were significantly higher than those in the EM group at the six sampling periods, whereas 16 bacterial biomarkers in the EM group were significantly higher than those in the M group.

### 3.4. Associations between water quality and bacterial communities

**Figure 9:**
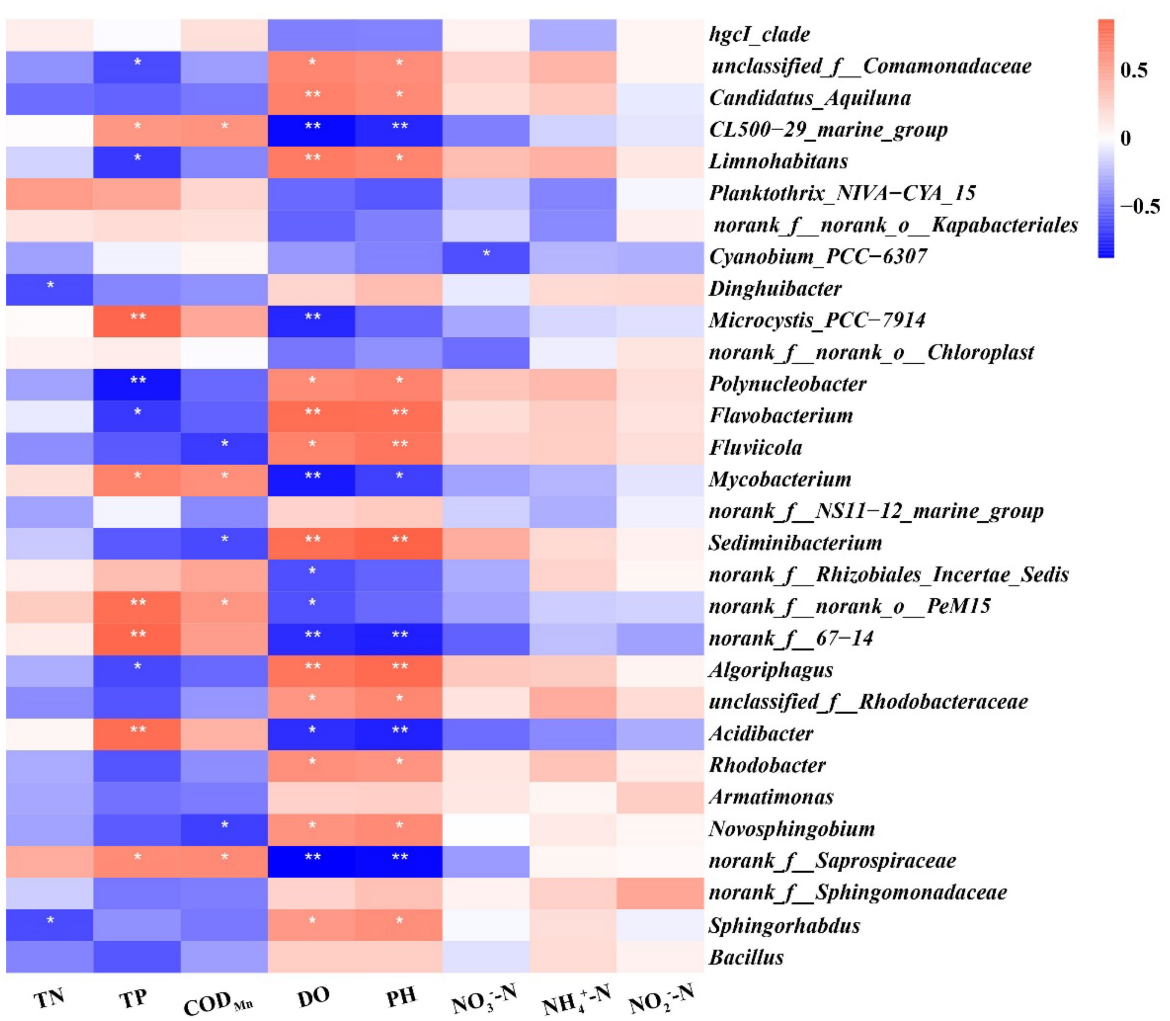
Correlation heatmap between eight physicochemical parameters and 30 important genera with differential abundance. Different shades of colors represent correlation level, with lighter shades indicating lower correlation and darker shades indicating higher correlation; red represents positive correlation and blue represents negative correlation. *p < 0.05; **p < 0.01.

A Spearman’s correlation analysis was performed to evaluate the potential associations between water physicochemical parameters and bacterial communities (Figure 9). We found that more predominant genera, including *unclassified_f__Comamonadaceae, Candidatus_Aquiluna, Limnohabitans, Polynucleobacter, Flavobacterium, Fluviicola, Sediminibacterium, Algoriphagus, unclassified_f__Rhodobacteraceae, Rhodobacter, Novosphingobium*, and *Sphingorhabdus*, were positively correlated with DO and pH (*p* < 0.05), whereas *CL500-29_marine_group, Mycobacterium, norank_f__67–14*, *Acidibacter*, and *norank_f__Saprospiraceae* were negatively correlated with DO and pH (*p* < 0.05). Furthermore, *Microcystis_PCC-7,914*, *norank_f__norank_o__PeM15*, *norank_f__67–14*, and *Acidibacter* exerted very significant positive correlations with TP (*p* < 0.01), which can be used as an indicator of trophic status. In addition, *Dinghuibacter* and *Sphingorhabdus* were found to be significant negatively correlated with TN (*p* < 0.05).

## 4. Discussion

Intensive aquaculture is on the rise worldwide and includes introducing formulated feed into production systems. However, in most countries, unstable and uncontrolled traditional intensive production has led to environmental and social problems (Rabalais et al., 2010; Troell et al., 2014; Fu, 2016; Waajen et al., 2016; Paerl, 2017; Jose Huertas and Mallen-Ponce, 2022). Given the negative impacts of intensive aquaculture, fishermen and scientists have pushed for more new approaches to fisheries practices based on ecosystem function and biological regulation (Ding et al., 2021; Tang et al., 2021).

This study is the first attempt to use constructed ecological ponds of cultured largemouth bass with submerged macrophytes, and was conducted because numerous studies have confirmed that macrophytes have prominent role in regulating water quality (Liu H. et al., 2020; Xia et al., 2020). Specifically, the TN and TP concentrations in aquaculture water can be significantly reduced through adsorption by aquatic plants (Ding et al., 2020; Zhang et al., 2021), which is consistent with the results of this experiment. High concentrations of TN and TP are an essential indicator of water eutrophication (Subbiah et al., 2019), and it is generally accepted that climate warming and eutrophication exacerbate phytoplankton blooms in water bodies (Amorim and Moura, 2021). In this experiment, Cyanobacterial outbreaks occurred in the M group ponds from day 7 to the end of the experiment; however, no Cyanobacterial blooms occurred in the EM group ponds throughout the culture period. Such results supported our prediction that ecological aquaculture with submerged macrophytes would have a significant effect on preventing Cyanobacterial outbreaks by reducing TN and TP in water. In addition, COD_Mn_ is an indicator of organic pollution (Yin et al., 2011), and it was significantly higher in the M group ponds without submerged macrophytes in this experiment; this may have been due to the significantly larger number of phytoplankton. Cheng et al. (2010) also found that COD_Mn_ was positively correlated with the growth of phytoplankton.

Phytoplankton are single-celled organisms that are sensitive to environmental changes and can serve as excellent indicators. Variations in phytoplankton can reflect both the state of health of the aquaculture water conditions, and the structure and function of the aquatic ecosystem (Saifullah et al., 2016; Zhang et al., 2020). According to Palikova et al. (2007) and Drobac et al. (2016), enrichment of Cyanobacteria leads to harmful blooms in which aquatic animals can be exposed to Cyanobacteria and their secondary metabolism in various ways, which may affect their growth, development, histology, and survival. In aquatic systems, Cyanobacterial blooms are a common biological disturbance that affect aquatic ecosystems (Xue et al., 2018). To combat the issues generated by Cyanobacterial blooms, aquaculture primarily relies on the use of chemical controls owing to their effectiveness in rapidly reducing phytoplankton biomass (Barrington et al., 2013; Bishop and Richardson, 2018; Sinha et al., 2018). Nonetheless, there are concerns about the negative effects of chemicals on non-target organisms because of their prolonged persistence in the environment and the need for repeated applications to prevent bloom resurgence, which increase water quality management costs and toxicity risks (Viriyatum and Boyd, 2016; Buley et al., 2021).

In this paper, ecological aquaculture with submerged macrophytes significantly reduced the content of harmful Cyanobacteria, and there were no Cyanobacterial outbreaks during the cultivation period, which was in sharp contrast to the ponds without submerged macrophytes. The submerged macrophytes competed with phytoplankton to absorb excess nutrients and may have also inhibited phytoplankton growth by secreting allelochemicals (Vanderstukken et al., 2014). Erhard and Gross (2006) found that *Elodea nuttallii* abundance was inversely correlated with phytoplankton or epiphyte coverage, which indicated that the hydrophilic and slightly lipophilic compounds extracts of *E. nuttallii* were responsible for reduction of Cyanobacterial growth. Therefore, submerged plants have a positive and stable effect on phytoplankton characteristics in aquaculture ecosystems.

The diversity and stability of bacterial community structure plays a vital role in the health and growth of aquatic animals (Zeng et al., 2019). Previous studies showed that the dominant phyla of water bacteria in largemouth bass (Liu Z. et al., 2020) and *Litopenaeus vannamei* (Zhang et al., 2016) ponds are Proteobacteria, Cyanobacteria, Actinobacteria, and Bacteroidetes, which were consistent with the results of this experiment. However, in this study, we observed that Proteobacteria and Bacteroidetes were significantly enriched in the EM group, whereas Actinobacteria and Cyanobacteria were significantly enriched in the M group; this may be caused by submerged macrophytes, aquatic animals, and plants releasing nutrients into the water through excretion and defoliation, and plants can also provide attachment points for bacteria and promote the reproduction of plant-related bacteria. In addition, plants can inhibit the growth of some microorganisms by secreting allelopathic substances. Chao et al. (2021) found that proteobacteria accounted for a significantly higher proportion of the bacterial community during the restoration of submerged vegetation in shallow eutrophic lakes, whereas actinomycetes accounted for a significantly higher proportion in the bare zone. Van der Gucht et al. (2005) found that Actinobacteria and Cyanobacteria are the dominant bacteria in eutrophic ponds, and Cyanobacteria are prone to blooms in which they release toxins that harm aquatic organisms. Proteobacteria, including nitrifying bacteria, denitrifying bacteria, sulfur-reducing bacteria, are widely distributed in water bodies and play important roles in the cycles of carbon, nitrogen, phosphorus, and other nutrients (Zhang et al., 2015). Bacteroidetes are also a dominant bacterium in aquaculture systems that can effectively degrade dissolved organic matter in water (Cottrell and Kirchman, 2000).

Microbial communities change over time through the cyclical process of assembly and succession (Cheong et al., 2021). The microbial succession patterns of dominant bacteria in this article showed stable differences between the two groups, and each group contained its own specific dominant bacterial biomarkers during the 90-day aquaculture period of largemouth bass. The dominant bacteria in the group without submerged macrophytes were mostly harmful bacteria, such as *Planktothrix_NIVA-CYA_15*, *Microcystis_PCC-7,914*, and *Mycobacterium*. Specifically, *Planktothrix_NIVA-CYA_15* and *Microcystis_PCC-7,914* belong to Cyanobacteria, and both can form toxic blooms in freshwater ecosystems that can harm aquatic life and, in severe cases, animal and human safety (Pancrace et al., 2017; Preece et al., 2017). *Mycobacterium*, as one of the most prevalent causes of chronic disease, affects freshwater fishes worldwide (Ravid-Peretz et al., 2019).

The EM group, which had submerged macrophytes, experienced significant enrichment of *Algoriphagus*, *Limnosphingobium*, *Polynucleobacter*, *unclassified_f__Comamonadaceae*, and *unclassified_f__Rhodobacteraceae*, which are functional bacteria involved in the nitrogen cycle (Boeuf et al., 2013; Ahmad et al., 2021; Liu W. et al., 2021). In particular, the nitrification process of ammonia nitrogen is an key process in which aerobic bacteria participate. Research shows that dissolved oxygen can effectively strengthen the growth of some aerobic functional bacteria. The results of correlation analysis are the same as those of this study. Dissolved oxygen significantly promotes the expansion of some nitrogen functional bacteria and promotes the cycle of nitrogen in water. In an environment with sufficient dissolved oxygen, ammonia nitrogen in water is easier to decompose into a state that does not pollute water. Moreover, there was enrichment of *Limnosphingobium*, *Novosphingobium*, *Candidatus_Aquiluna*, *Fluviicola*, *Rhodobacter*, and *Spingorhabdus*, which can enrich organic carbon and have great degradation potential for organic matter; some of them can even use organic matter as a carbon source to carry out photosynthetic reactions, thereby reducing the COD value in aquaculture water (Notomista et al., 2011; Kang et al., 2012; Zeng et al., 2012; Zhou et al., 2016; Woo et al., 2021). *Sediminibacterium* can produce compounds such as glucose in bacteria to provide materials needed for metabolism of other microorganisms (Kim et al., 2009). There was also enrichment of *Bacillus*, which is a probiotic for animals and plants that can reduce ammonia and nitrite in water, colonize plant roots to promote plant growth, and use various mechanisms to promote the intestinal health of animals and regulate animal immunity; consequently, it is often used as a feed additive in aquaculture (Hoa et al., 2000; Ali et al., 2009; Yang et al., 2011; Guo et al., 2016). For example, Liu et al. (2012) found that adding *B. subtilis* to feed effectively improved the growth and immunity of *Epinephelus coioides*. *Flavobacterium* was also enriched and can use Cyanobacteria as a food source, which can inhibit the cell proliferation of Cyanobacteria; therefore, *Flavobacterium* has a good inhibitory effect on Cyanobacteria (Gerphagnon et al., 2015).

Overall, aquatic plants and their bacterial communities strongly interact in aquatic systems (Hempel et al., 2008; Xia et al., 2020), which was supported by the results of this paper. This interaction was positive, beneficial, and consistent, which indicates that the planting of submerged macrophytes produced a valuable relationship with bacteria and contributed to maintaining aquaculture ecosystem stability.

## 5. Conclusion

Aquaculture with submerged macrophytes significantly decreased the concentrations of TP, TN, and COD_Mn_, but significantly increased DO concentrations. These changes caused by submerged macrophytes had positive and stable effects on the phytoplankton and microbial communities. In particular, the M group had higher phytoplankton density and mainly included Cyanobacteria, whereas the EM group had lower phytoplankton density and mainly included Chlorophyta. Moreover, the alpha diversity of bacterial communities was influenced, with higher ACE index values observed in the EM group compared with the M group. Furthermore, PCoA clearly grouped the bacterial communities according to the two culture modes throughout the culture period; most bacteria in the M group were harmful to aquatic organisms, whereas those of the EM group are generally considered beneficial functional bacteria because of their involvement in material circulation and immune function. Therefore, aquaculture systems that include submerged macrophytes can improve water quality and control of Cyanobacterial blooms, and affect bacterial community diversity and composition. What’s more, the fish performed better in the submerged macrophyte than the no macrophyte ponds when stocked at similar densities. These effects seem to be beneficial and consistent, and this is an environmentally friendly and high value-added ecological aquaculture model worthy of extensive application and popularization.

## Data availability statement

The datasets presented in this study can be found in online repositories. The names of the repository/repositories and accession number (s) can be found in the article.

## Ethics statement

The animal study was reviewed and approved by Freshwater Fisheries Research Center (FFRC), Chinese Academy of Fishery Sciences (CAFS).

## Author contributions

GX: accepted and designed research, reviewed the manuscript and approved the final manuscript. PX: reviewed the manuscript and approved the final manuscript. ZN: designed research, guided experimental methodology, wrote the manuscript, reviewed the manuscript and approved the final manuscript. ZZ: feeding, sampling, conducting the experiment, data analysis, wrote the manuscript and approved the final manuscript. HZ: sampling, collected and analyzed the data, read and approved the final manuscript. YS: sampling, feeding and approved the final manuscript. JG and JcG: sampling, data analysis, approved the final manuscript. All authors contributed to the article and approved the submitted version.

## Funding

This work was supported by National Key Research and Development Program of China (No. 2019YFD0900301), and the earmarked fund for CARS-46.

## Conflict of interest

The authors declare that the research was conducted in the absence of any commercial or financial relationships that could be construed as a potential conflict of interest.

## Publisher’s note

All claims expressed in this article are solely those of the authors and do not necessarily represent those of their affiliated organizations, or those of the publisher, the editors and the reviewers. Any product that may be evaluated in this article, or claim that may be made by its manufacturer, is not guaranteed or endorsed by the publisher.

## Footnotes

^1^ https://cloud.majorbio.com

^2^ http://huttenhower.Sph.harvard.edu/LEfSe
